# Sodium fluoride exposure exerts toxic effects on porcine oocyte maturation

**DOI:** 10.1038/s41598-017-17357-3

**Published:** 2017-12-06

**Authors:** Shuang Liang, Zheng-Wen Nie, Minghui Zhao, Ying-Jie Niu, Kyung-Tae Shin, Xiang-Shun Cui

**Affiliations:** 1 Department of Animal Science, Chungbuk National University, Cheongju, Chungbuk 361-763 Republic of Korea; 2 Department of Animal Sciences, College of Animal Sciences, Jilin University, Changchun, 130062 China

## Abstract

Excessive long-term fluoride intake is associated with several health problems, including infertility. However, limited information is available on the toxic effects of fluoride exposure on the female reproductive system, especially oocyte maturation. In this study, we investigated the toxic effect of sodium fluoride (NaF) exposure on porcine oocyte maturation and its possible underlying mechanisms. Our results showed that NaF exposure during porcine oocyte maturation inhibited cumulus cell expansion and impaired polar body extrusion. Cell cycle analysis showed that NaF exposure blocked meiotic resumption, disturbed spindle dynamics, disrupted chromosome separation, and increased aneuploidy in porcine oocytes. Moreover, NaF exposure disturbed mitochondrial function, triggered DNA damage response, and induced early apoptosis in porcine oocytes. NaF exposure also induced oxidative stress, decreased GSH level, and increased cathepsin B activity in and impaired the further development potential of porcine oocytes, as indicated by a decrease in blastocyst formation rate, increase in apoptosis, and inhibition of cell proliferation. Together, these results indicate that NaF exposure impairs the maturation capacity of porcine oocytes by inhibiting cumulus cell expansion, disturbing cytoskeletal dynamics, and blocking nuclear and cytoplasmic maturation, thus decreasing the quality and affecting the subsequent embryonic development potential of porcine oocytes.

## Introduction

Fluorine is a common element that does not occur in the nature in its elemental state because of its high reactivity. It accounts for approximately 0.3 g/kg of the earth’s crust and exists in the form of fluorides in several minerals. Fluoride is an inorganic, monatomic anion of fluorine. Fluoride salts and minerals are important chemical reagents for producing fluorocarbons in industry. In the nature, fluorine is widely distributed in seawater and underground water^1^. Concentration of fluoride in groundwater depends on the composition of local rocks and ranges between 0.05 and 8 mg/L^2,3^.

Exposure to small amounts of fluoride is beneficial to human health, e.g., small amounts of fluoride are used to prevent dental caries and to regulate the growth and development of skeletal tissue. The World Health Organization has suggested that fluoride concentration in water should be 1.5 mg/L^4^. A concentration above this value is associated with increased risk of dental fluorosis, and much higher concentrations are associated with skeletal fluorosis. Excessive fluoride intake exerts negative effects on the human body. Several experimental and clinical studies have suggested that excessive fluoride intake results in fluoride accumulation in the bones, teeth, and other tissues and organs, resulting in physiological dysfunction, including mutagenesis, immune suppression, carcinogenesis, and growth retardation^5–8^. The developing brain is one of the major targets of fluoride. Results of epidemiological studies have shown that children residing in high-fluoride areas have significantly lower intelligence quotient (IQ) scores than those residing in low-fluoride areas^9^. Treatment of rats with high fluoride concentration induces S phase cell cycle arrest, DNA damage, and NF-kappa B upregulation in hippocampal neurons^10^. Fluoride readily crosses the placenta, and exposure of the developing brain, which has increased susceptibility to injury, to fluoride may permanently damage the fetus^11^. Other studies have shown that fluoride intake through drinking water induces liver damage by markedly increasing aspartate transaminase and alanine transaminase levels and promotes lipid peroxidation in liver cells in mice and their pups^12^.

Several studies have assessed the toxicity of fluoride on the male reproductive system of rodents and humans residing in fluorosis endemic areas^13,14^. Animal studies have shown that fluoride negatively affects the male reproductive system by injuring sperm function, including its morphology, motility, capacitation, and acrosome reaction^13,15,16^. Fluoride also disturbs Fas/Fas ligand system, thus suppressing the immune privileged function of mouse Sertoli cells^17^. Rabbits fed a fluoride-rich diet for 60 days show significant atrophy and necrosis in the seminiferous tubules and abnormalities in all spermatocytes or spermatids^18^. In addition, consumption of drinking water containing high fluoride content is associated with low birth rates in humans^15^. Our previous study showed that consumption of fluoride-rich diet exerts dose-dependent effects on mouse oocyte maturation, with high fluoride concentration inducing abnormal spindle formation and mitochondrial dysfunction in mouse oocytes matured *in vivo* ^19^. Furthermore, fluoride administration hampers the development of and affects epigenetic modification in early mouse embryos^20,21^. Rats exposed to increasing sodium fluoride (NaF) doses show a marked decrease in successful pregnancy rates because of a decrease in the secretion of steroid hormones progesterone and estrogen and increase in abnormal follicular development^22^.

Although several studies have investigated the negative effect of fluoride on the male and female reproductive system, its underlying mechanism of action is unclear. Moreover, differences in physiology and metabolism between rodents and humans have resulted in the wastage of medical resources^23^. Compared with rodents, pigs are more similar to humans, which has more benefits in researches of human reproduction^24,25^. In addition, pig oocyte maturation period is longer than that of the used rodents model^26^, which provides an opportunity to investigate in more detail the mechanism of oocyte maturation. Oocyte quality is a major factor in reproduction. Poor quality of oocytes hampers embryonic development after fertilization, thus affecting the health of the fetus and posterity. Studies on environmental toxicology have shown that toxic chemicals promote abnormal oocyte growth and embryonic development^27–29^.

However, no study has assessed the effect of fluoride exposure on the porcine reproductive system. Analysis of the effect of fluoride on porcine germ cell development will provide useful information for human biomedical research. Therefore, the present study evaluated the effect of fluoride on porcine oocyte maturation and explored the underlying mechanism at a subcellular level.

## Results

### NaF exposure during IVM impairs cumulus cell expansion and polar body extrusion in porcine oocytes

**Figure 1:**
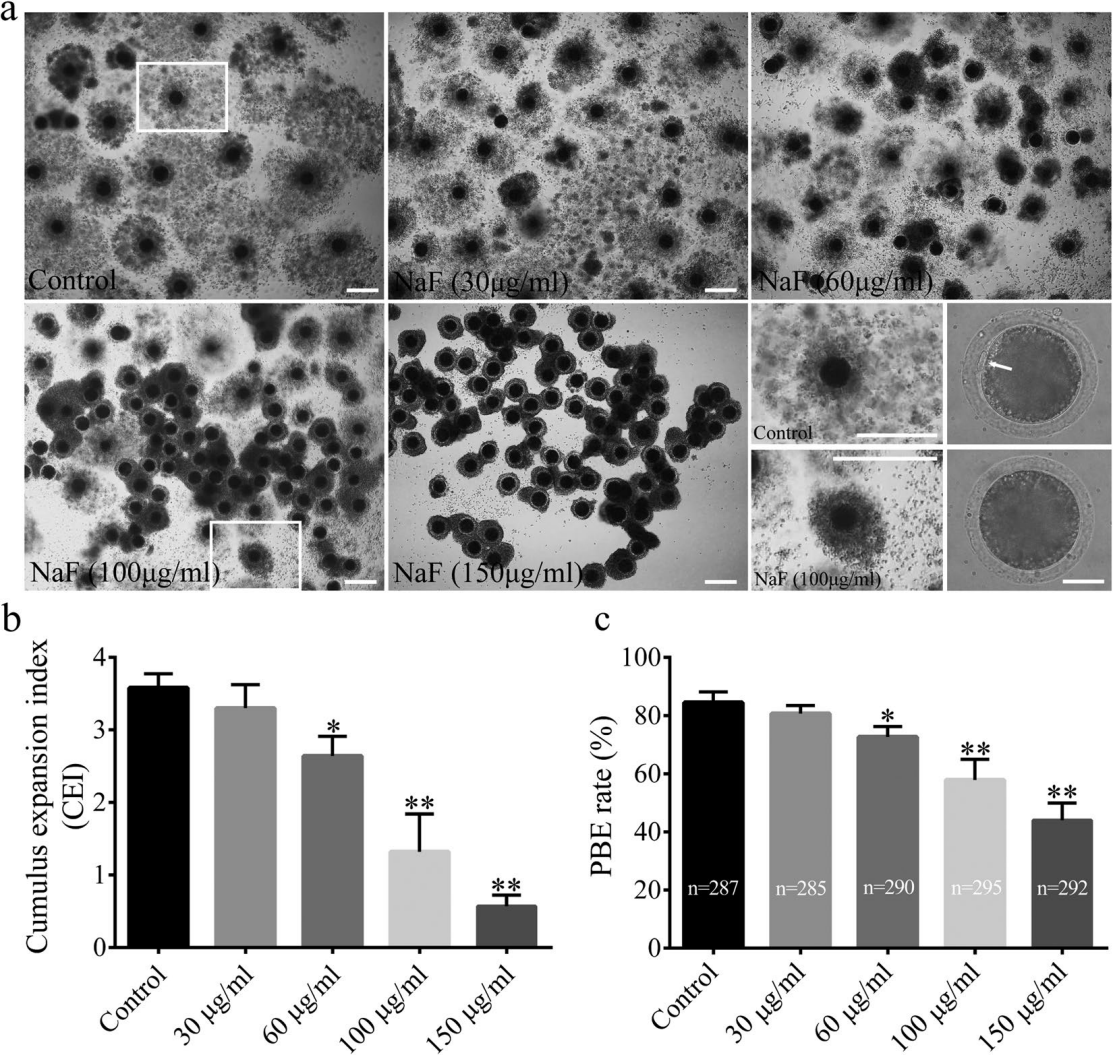
Effect of NaF exposure on cumulus cell expansion and PBE rate in porcine oocytes. (**a**) COCs were cultured with 30, 60, 100, and 150 μg/ml NaF for 44 h. Cumulus cell expansion decreased after NaF exposure; COCs: scale bar = 200 μm; oocyte: scale bar = 50 μm. (**b**) Average CEI was calculated at 44 h for COCs in each experimental group. (**c**) PBE rate decreased significantly after NaF treatment. Data are presented as mean ± SD of three independent experiments. Statistically significant differences are indicated by asterisks (*p < 0.05 and **p < 0.01).

To determine whether NaF exposure affected porcine oocyte maturation *in vitro*, COCs were incubated with increasing concentrations of NaF (30, 60, 100, and 150 μg/ml) and the degree of cumulus cell expansion and rate of maturation were analyzed. Treatment of the oocytes with 30 μg/ml NaF did not affect the degree of cumulus cell expansion (CEI: 3.30 ± 0.32 vs. 3.58 ± 0.19, p > 0.05) and the rate of maturation (80.67% ± 2.73% vs. 84.50% ± 3.62%, p > 0.05). However, treatment with 60, 100, and 150 μg/ml NaF significantly decreased the degree of cumulus cell expansion (CEI: 2.64 ± 0.27, 1.32 ± 0.52, and 0.57 ± 0.15 vs. 3.58 ± 0.19, p < 0.05) and the rate of maturation (74.33% ± 4.32%, 57.83% ± 7.14%, 44.00% ± 5.93% vs. 84.50% ± 3.62%, p < 0.05) in a dose-dependent manner (Figs. 1 and S1). These results suggest that NaF exposure suppressed cumulus cell expansion and polar body extrusion (PBE) in porcine oocytes in a dose-dependent manner. Therefore, 100 μg/ml NaF was used for performing all subsequent experiments.

### NaF exposure triggers DNA damage and apoptosis in porcine cumulus cells

**Figure 2:**
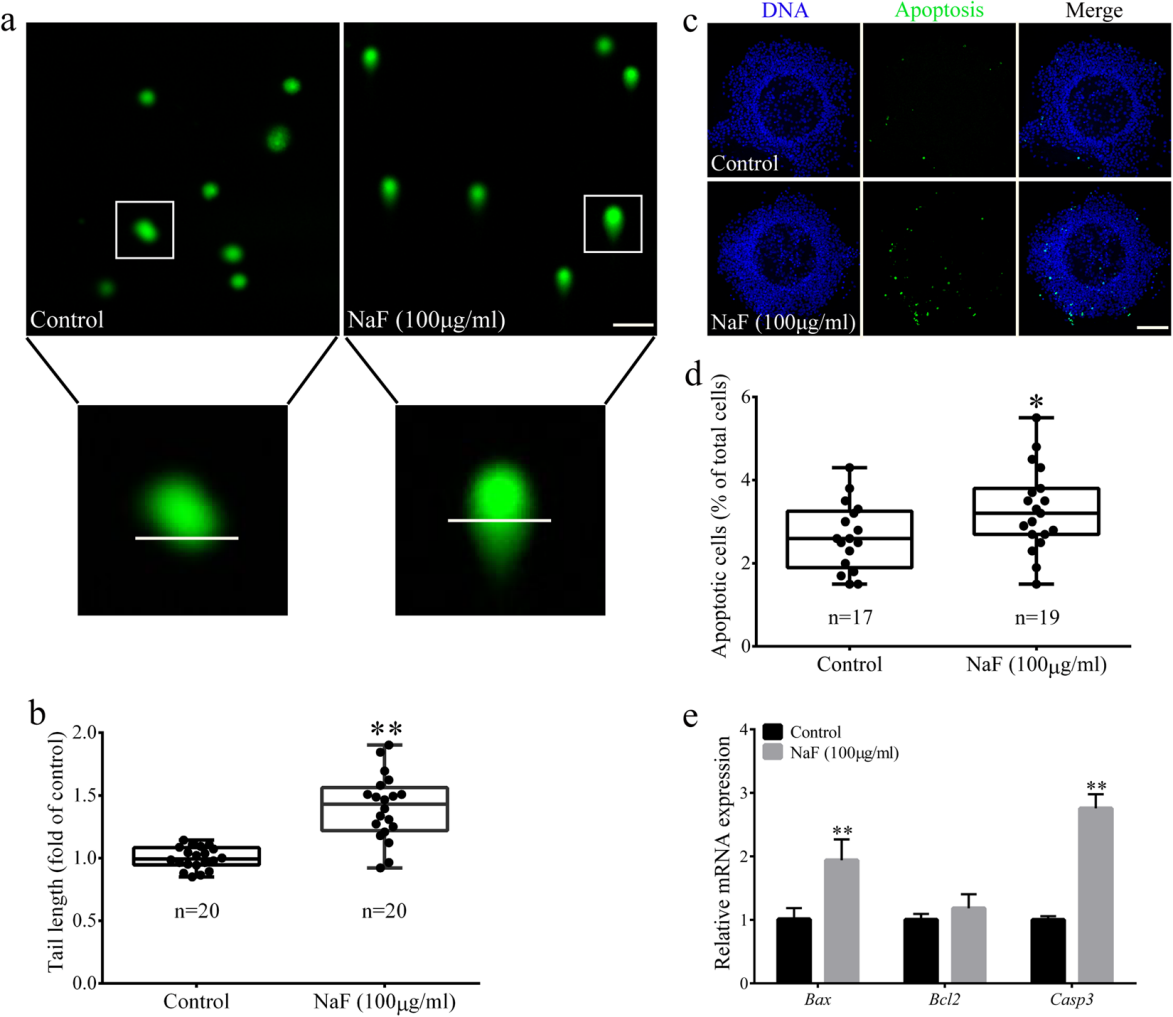
NaF exposure induces DNA damage and apoptosis in porcine cumulus cells. (**a**) DNA damage in cumulus cells was assessed by performing the comet assay. Control cumulus cells showed slight DNA damage, whereas NaF-treated cumulus cells showed notable DNA damage. (**b**) Fold changes in tail moment and length in NaF-treated cumulus cells. (**c**) Apoptosis of cumulus cells was assessed by performing the TUNEL assay. The percentage of TUNEL-positive apoptotic cumulus cells significantly increased after NaF treatment. (**d**) Quantitative analysis of the effect of NaF on the apoptosis of cumulus cells; scale bar = 50 μm. (**e**) Expression of apoptosis-related genes in cumulus cells. Data are presented as mean ± SD of three independent experiments. Statistically significant differences are indicated by asterisks (*p < 0.05 and **p < 0.01).

NaF induces DNA damage and apoptosis in mammalian cells. Therefore, we determined whether NaF treatment induced DNA damage and apoptosis in porcine cumulus cells. Results of DNA comet assay showed that DNA damage markedly increased in NaF-treated oocytes compared with that in control oocytes (p < 0.01; Fig. 2a,b). Furthermore, the proportion of TUNEL-positive apoptotic cells significantly increased among NaF-treated COCs compared with that among control COCs (p < 0.05; Fig. 2c,d). Moreover, NaF treatment increased the expression of apoptotic genes *BAX* and *CASP3* (p < 0.01; Fig. 2e). These data suggest that NaF exposure induces DNA damage and apoptosis in porcine cumulus cells, which in turn affects oocyte maturation.

### NaF exposure disturbs meiotic cell cycle, induces spindle defects, affects spindle position, and induces aneuploidy during porcine oocyte meiotic maturation

**Figure 3:**
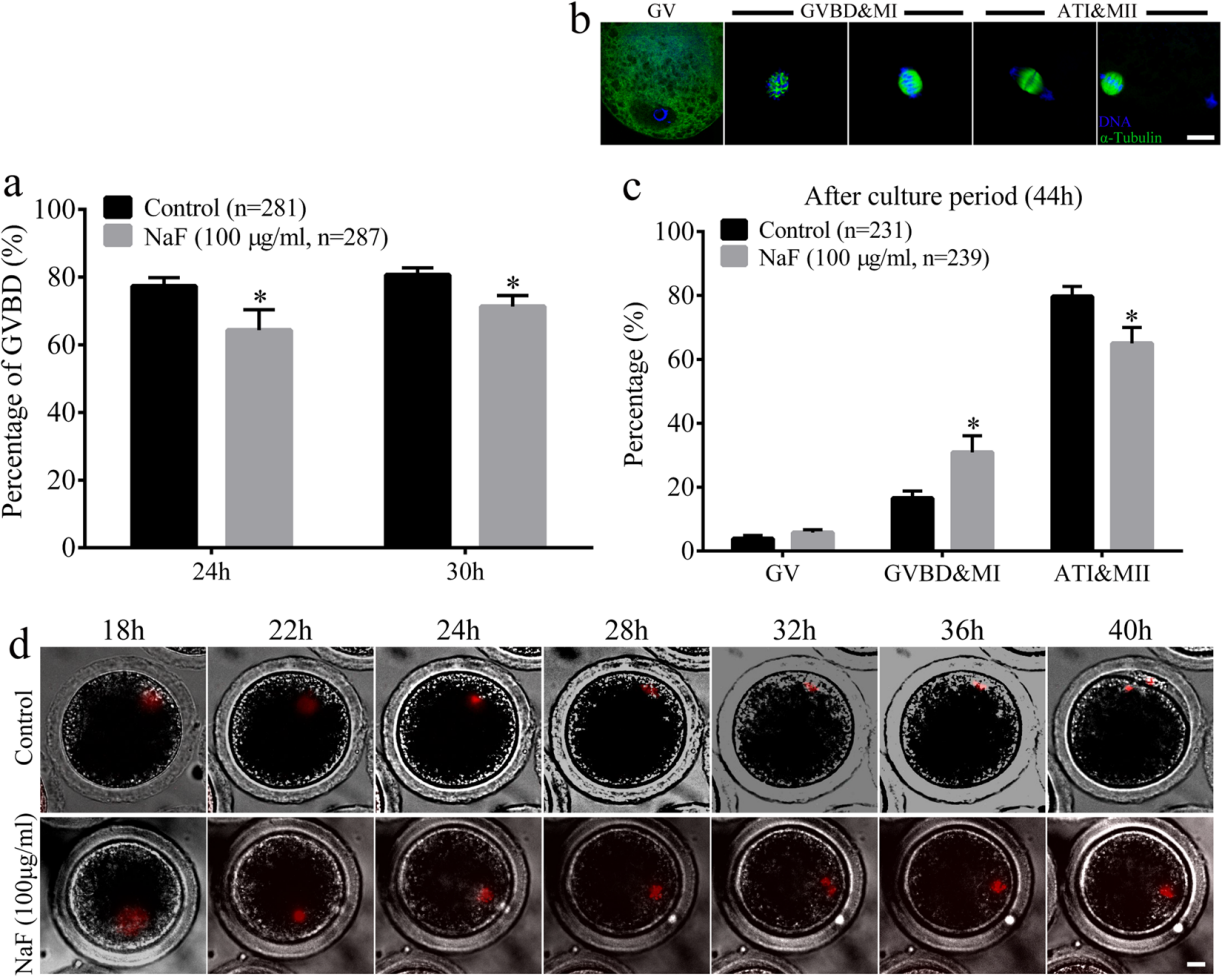
NaF exposure affects cell cycle progression in porcine oocyte. (**a**) The percentage of oocytes undergoing GVBD after NaF treatment for 24 or 30 h. (**b**) Representative confocal images of porcine oocytes in various maturation phases; scale bar = 10 μm; blue, DNA; green, α-tubulin. (**c**) Percentages of cells in various meiotic maturation phases were calculated at 44 h. (**d**) Time-lapse images obtained after microinjecting GV-stage oocytes with histone H2B–mCherry cRNA; Scale bar = 20 μm. ATI: anaphase/telophase I; GV: germinal vesicle; GVBD: germinal vesicle breakdown; MII: metaphase II. Data are presented as mean ± SD of three independent experiments. Statistically significant differences are indicated by asterisks (*p < 0.05).

The above results led us to hypothesize that NaF exposure affected the assembly of meiotic apparatus in porcine oocytes. To determine the possible mechanism underlying maturation failure, we first tracked meiotic resumption in porcine oocytes at different time points (Fig. 3a). After culturing for 24 and 30 h, germinal vesicle breakdown (GVBD) was significant reduced in NaF-treated oocytes (64.33% ± 6.03% and 71.33% ± 3.22%, respectively) compared with that in control oocytes (77.33% ± 2.52% and 80.67% ± 2.08%, respectively, p < 0.05). We next analyzed the percentage of oocytes arrested at different meiotic stages at 44 h. We found that most control oocytes were arrested in the anaphase/telophase I (ATI) and metaphase II (MII) stages, whereas most NaF-treated oocytes were arrested in the GVBD and metaphase I (MI) stages (Fig. 3b,c). Higher percentage of control oocytes were in the ATI/MII stage than NaF-treated oocytes (79.67% ± 3.21% vs. 65.00% ± 5.00%, p < 0.05). However, significantly higher percentage of NaF-treated oocytes were in the GVBD/MI stage than control oocytes (16.50% ± 2.29% vs. 30.90% ± 5.15%, p < 0.05). The percentage of oocytes in the germinal vesicle (GV) stage was not significantly different between control and NaF-treated oocytes (3.83% ± 1.04% vs. 5.77% ± 0.93, p > 0.05). Time-lapse microscopy was performed to confirm the negative effects of NaF on porcine oocyte meiotic maturation. The oocytes were microinjected with H2B–mCherry complementary RNA (cRNA) to evaluate chromatin movement. Control oocytes microinjected with H2B–mCherry cRNA reached the MII stage at 40 h, whereas NaF-treated oocytes microinjected with H2B–mCherry cRNA did not reach the MII stage at 40 h (Fig. 3d and Supplementary videos S1 and S2).

**Figure 4:**
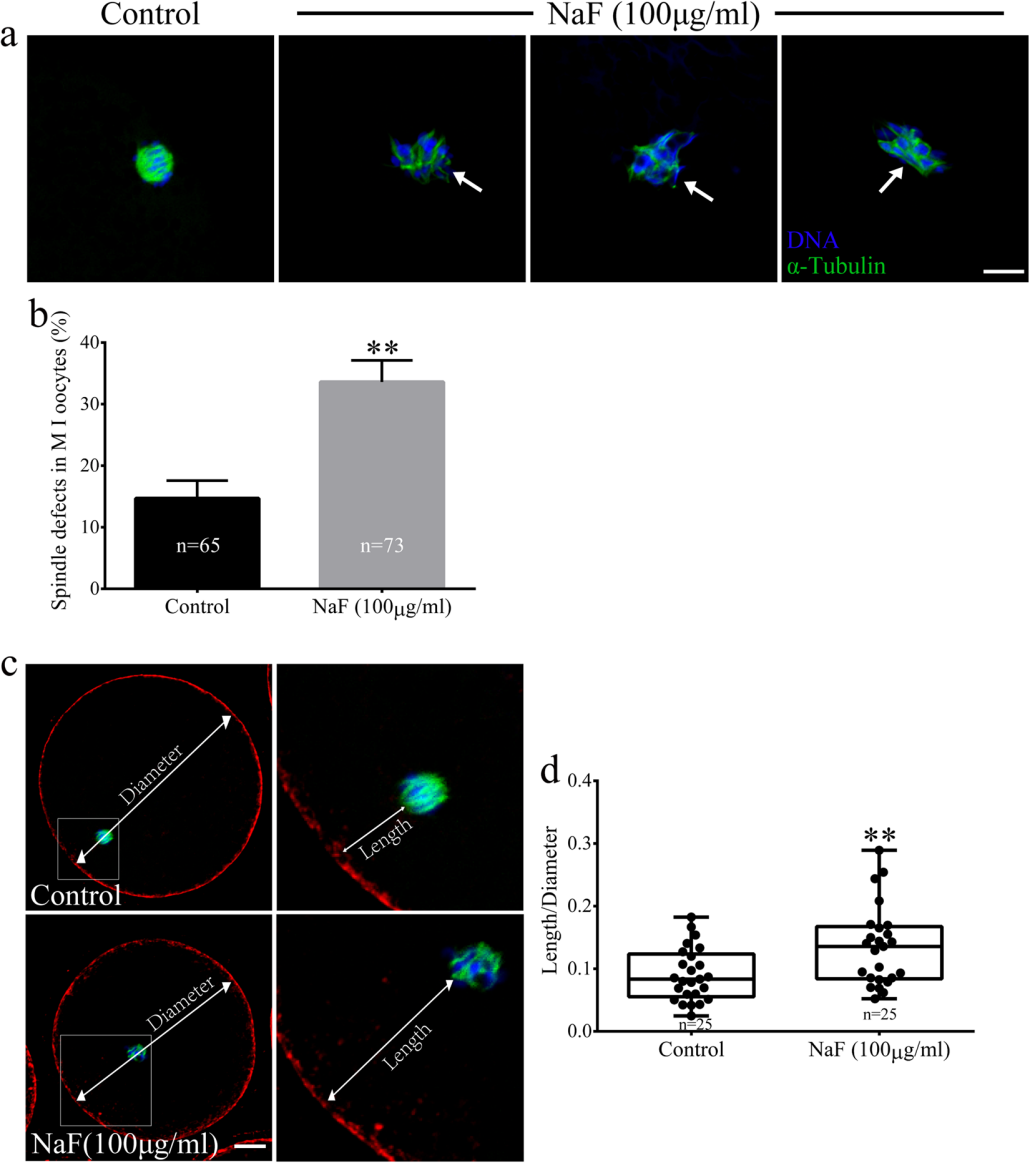
NaF exposure induces defects in spindle assembly in porcine oocytes. (**a**) Representative spindle morphologies of oocytes in the MI stage. Control oocytes showed normal spindle morphology, whereas NaF-treated oocytes showed various spindle defects (arrows); scale bar = 10 μm; blue, DNA; green, α-tubulin. (**b**) Percentages of NaF-treated oocytes in the MI stage showing aberrant spindle morphology. (**c**) Spindle positioning in oocytes in the MI stage. Spindles in control oocytes were located almost peripherally, whereas those in NaF-treated oocytes were located almost centrally; scale bar = 20 μm. (**d**) Quantification of the distance between the spindle and cell cortex (length/diameter) in control and NaF-treated oocytes. Data are presented as mean ± SD of three independent experiments. Statistically significant differences are indicated by asterisks (**p < 0.01).

**Figure 5:**
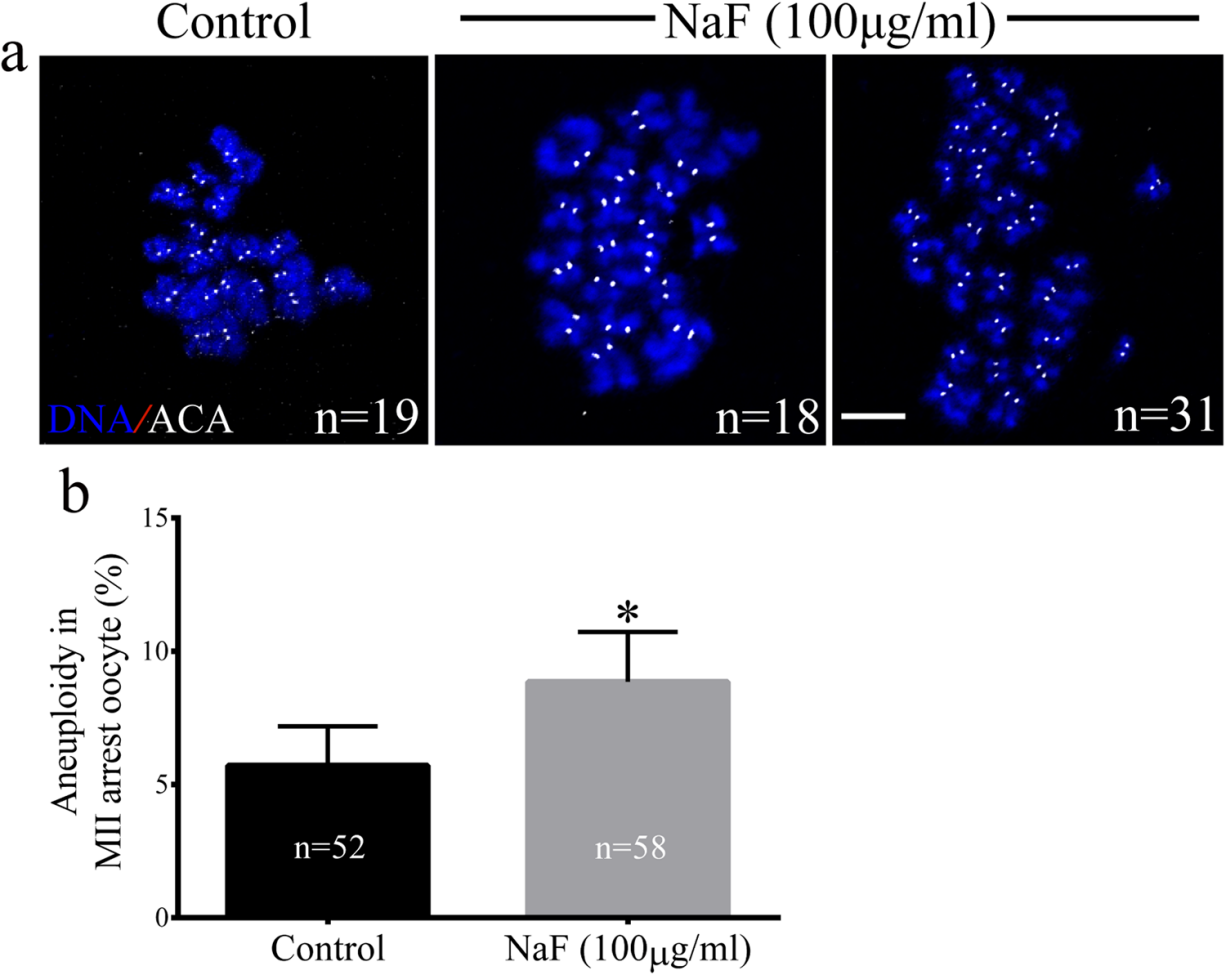
(**a**) Chromosome spreads of oocytes in the MII stage; scale bar = 10 μm; blue, DNA; white, kinetochore. (**b**) Quantification of aneuploidy in control and NaF-treated oocytes. Data are presented as mean ± SD of three independent experiments. Statistically significant differences are indicated by asterisks (*p < 0.05).

We next assessed the morphologies of meiotic spindles in NaF-treated oocytes in the MI and MII stages. For this, control and NaF-treated oocytes in the MI and MII stages were immunolabeled with anti-α-tubulin antibody to visualize the meiotic spindles and were counterstained with Hoechst 33342 to label chromosomes. Control oocytes showed normal spindle morphology, whereas NaF-treated oocytes showed disrupted spindle morphology (Fig. 4a). Significantly higher percentage of NaF-treated oocytes in the MI stage showed abnormal spindles than control oocytes in the MI stage (14.70% ± 2.89% vs. 33.60% ± 3.51%, p < 0.05; Fig. 4b). We next assessed spindle positioning in the oocytes. We measured the distance between the spindle and cell cortex (Fig. 4c) and found that the average distance (length/diameter) was significantly higher in NaF-treated oocytes than in control oocytes (0.09 ± 0.04 vs. 0.14 ± 0.06, p < 0.05; Fig. 4d). Next, we investigated the ploidy status of MII-stage oocytes by preparing chromosome spreads and by labeling kinetochores (Fig. 5a). We found that significantly higher percentage of NaF-treated oocytes showed aneuploidy than control oocytes (5.71% ± 1.47% vs. 8.56% ± 1.86%, p < 0.05; Fig. 5b). These results suggest that NaF exposure blocks cell cycle progression and disrupts spindle assembly and chromosome separation during porcine oocyte meiotic maturation.

### NaF exposure affects mitochondrial function in porcine oocytes

**Figure 6:**
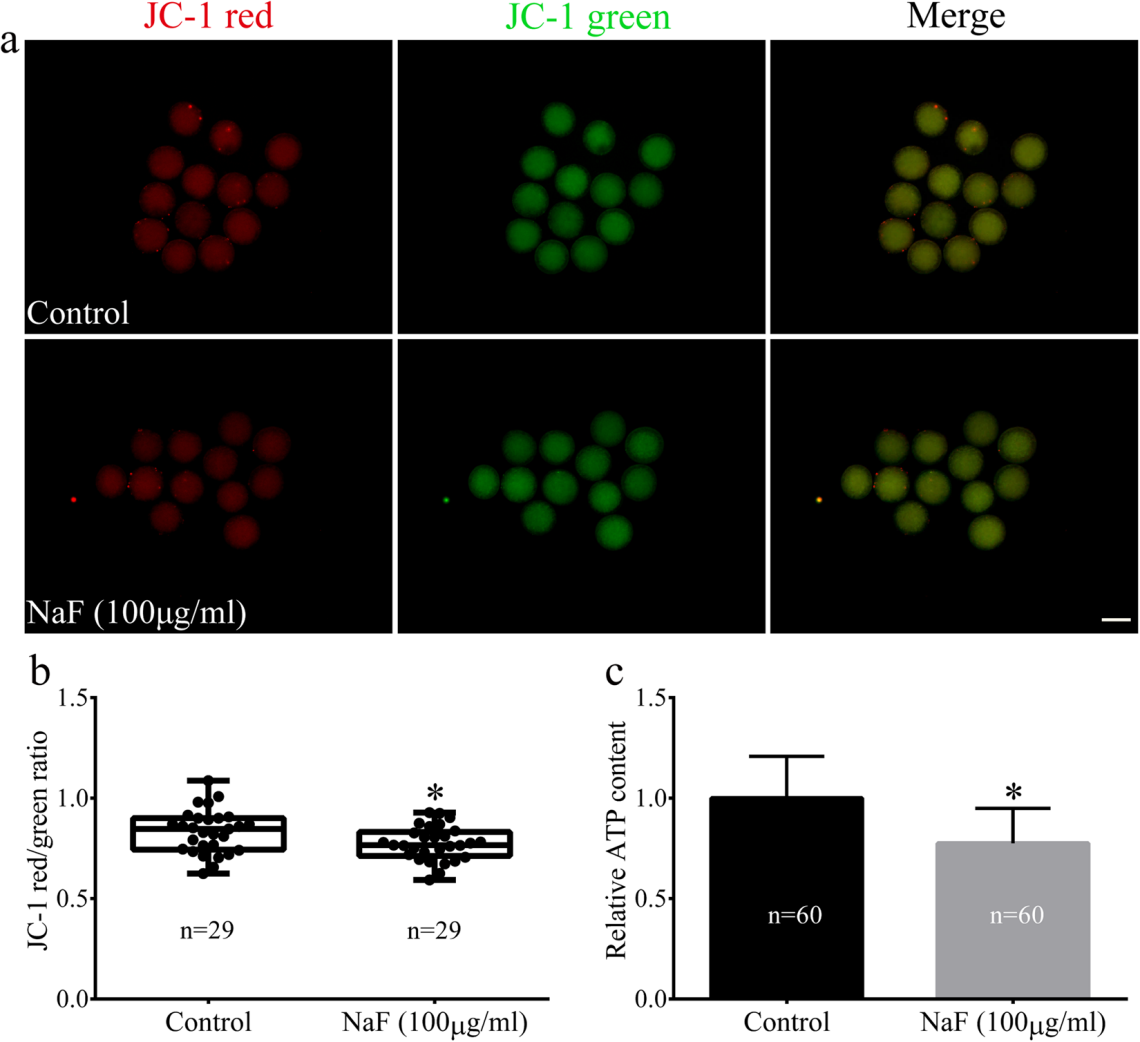
NaF exposure induces mitochondrial dysfunction (as determined by measuring ΔΨm and ATP production) in porcine oocytes. (**a**) JC-1 staining of NaF–treated oocytes. ΔΨm was significantly lower in NaF-treated oocytes than in control oocytes; scale bar = 100 μm. (**b**) Fluorescence intensity of JC-1 in oocytes. (**c**) Intracellular ATP content in oocytes. NaF exposure clearly affected mitochondrial ATP production. Data are presented as mean ± SD of three independent experiments. Statistically significant differences are indicated by asterisks (*p < 0.05).

The mitochondria of mammalian cells play a key role in producing cellular energy. Therefore, we determined whether NaF exposure affected mitochondrial function. For this, we evaluated ΔΨm and ATP production in porcine oocytes. Representative images of mitochondrial ΔΨm are shown in Fig. 6a. The average ΔΨm (Fig. 6b) and ATP production (Fig. 6c) decreased significantly in NaF-treated oocytes compared with that in control oocytes (p < 0.05). These results indicate that NaF exposure disturbs mitochondrial function in porcine oocytes.

### NaF exposure induces ROS formation, decreases GSH levels, and increases cathepsin B activity in porcine oocytes

**Figure 7:**
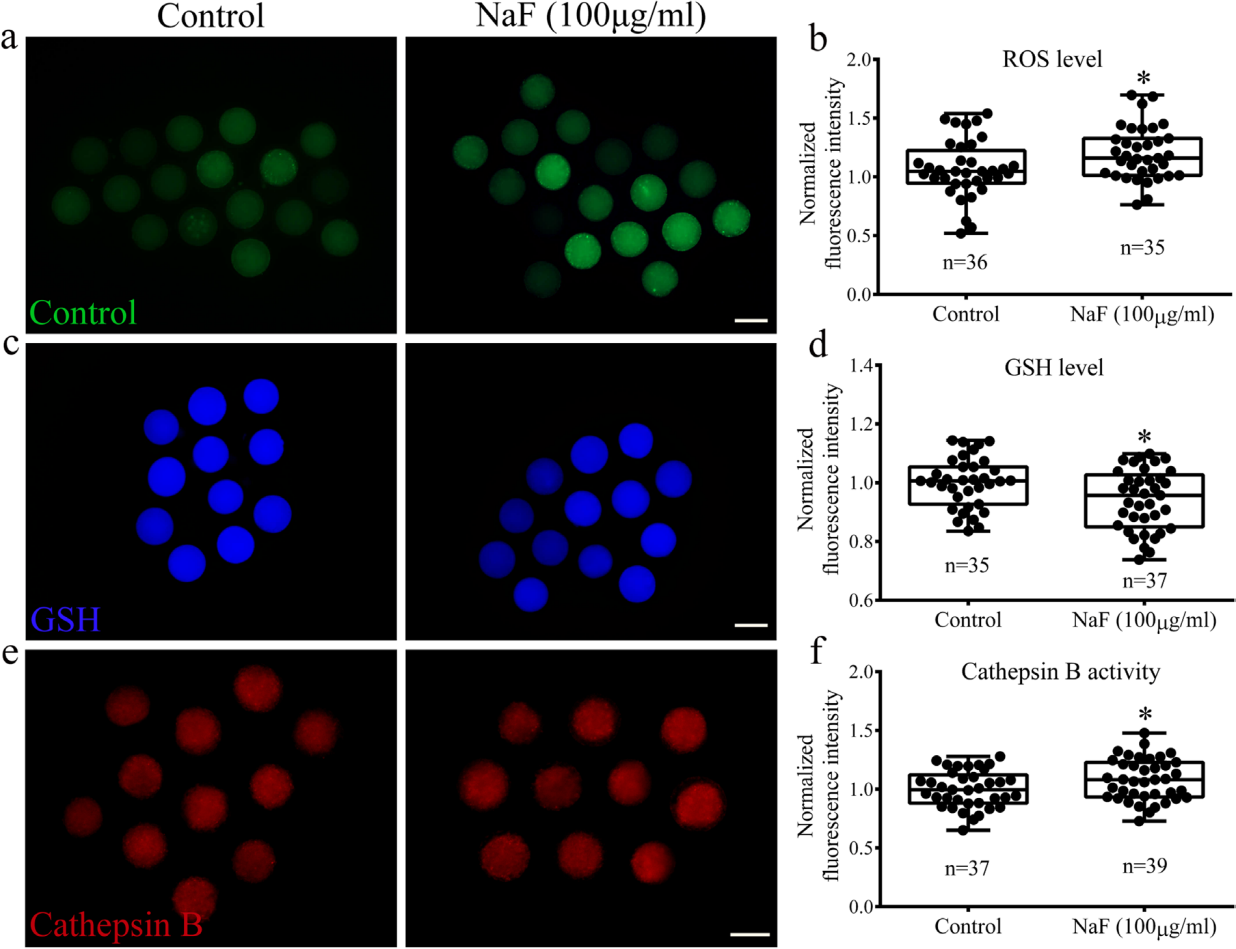
NaF exposure effects on ROS level, GSH level, and cathepsin B activity in porcine oocytes. (**a**) NaF exposure increased ROS generation. (**b**) Relative fluorescence intensity of ROS in control and NaF-treated oocytes. (**c**) NaF exposure decreased GSH level. (**d**) Relative fluorescence intensity of GSH in control and NaF-treated oocytes. (**e**) NaF exposure increased cathepsin B activity. (**f**) Relative fluorescence intensity of cathepsin B in control and NaF-treated oocytes. Scale bar = 100 μm. Data are presented as mean ± SD of three independent experiments. Statistically significant differences are indicated by asterisks (*p < 0.05).

To analyze the mechanism through which NaF affected porcine oocyte maturation, we examined ROS generation, GSH levels, and cathepsin B activity in NaF-treated oocytes. We first determined ROS levels in oocytes by performing DCFH fluorescent reaction. As shown in Fig. 7a, the relative fluorescence intensity of ROS was significantly higher in NaF-treated oocytes than in control oocytes (p < 0.05; Fig. 7b. Next, we determined GSH levels in porcine oocytes (Fig. 7c). Quantitative analysis showed that the relative fluorescence intensity of GSH was significantly lower in NaF-treated oocytes than in control oocytes (p < 0.05; Fig. 7d). Next, we evaluated cathepsin B activity in porcine oocytes (Fig. 7e) and observed a significant increase in activated cathepsin B levels in NaF-treated oocytes (p < 0.05; Fig. 7f).

### NaF exposure triggers DNA damage and early apoptosis in porcine oocytes

**Figure 8:**
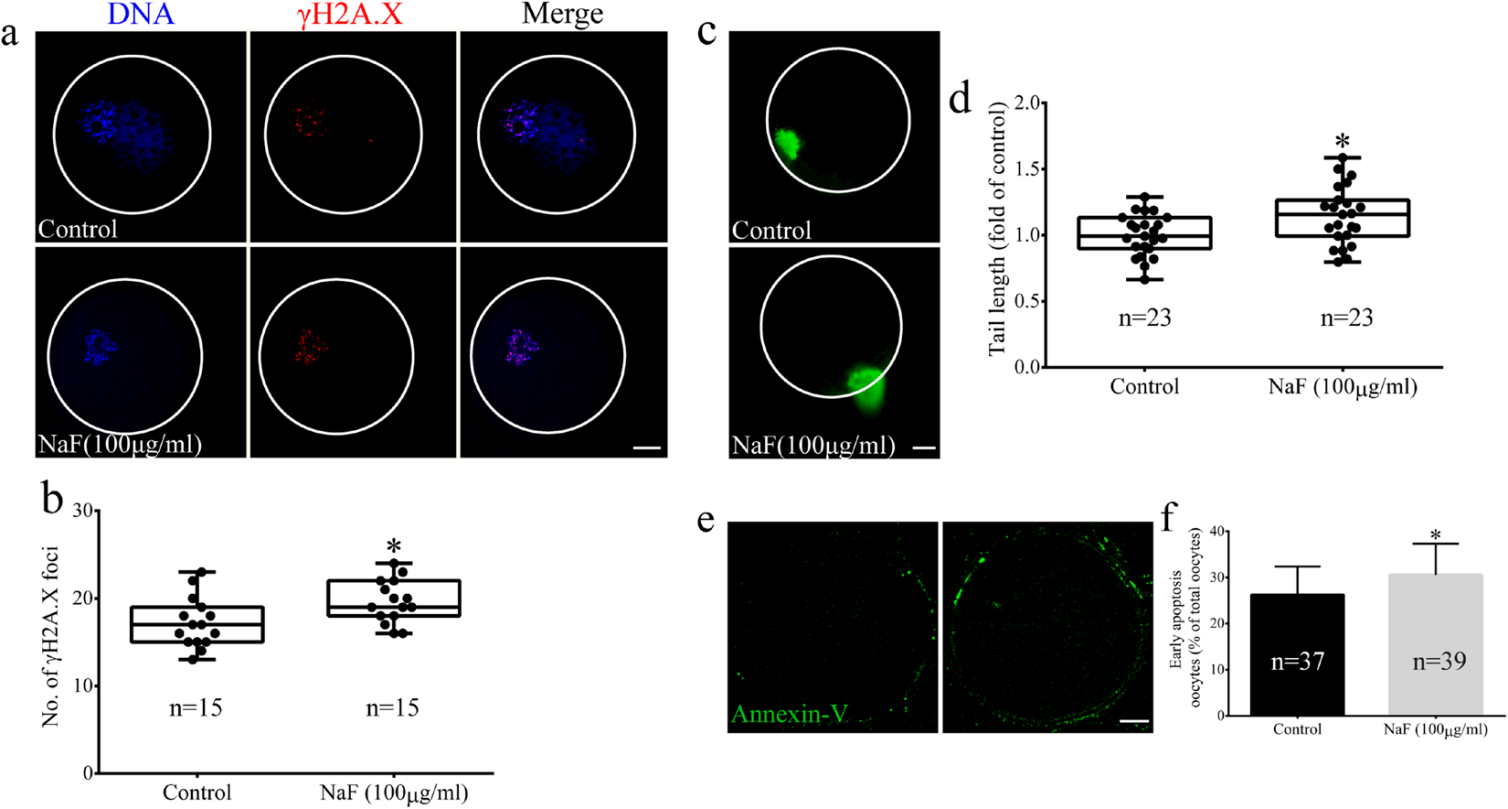
NaF exposure induces DNA damage and early apoptosis in porcine oocytes. (**a**) Localization of γH2A.X in the nuclei of oocytes. The number of γH2A.X foci significantly increased after NaF treatment; blue, DNA; red, γH2A.X. (**b**) Quantification of γH2A.X levels in the nuclei of control and NaF-treated oocytes. (**c**) DNA damage in oocytes was assessed by performing the comet assay. Control oocytes showed slight DNA damage, whereas NaF-treated oocytes showed notable DNA damage. (**d**) Fold changes in tail moment and length in NaF-treated oocytes. (**e**) Early apoptosis in oocytes was assessed by performing annexin V staining. NaF-treated oocytes showed fluorescent signals on the oocyte membrane, whereas control oocytes showed fluorescent signals on the zona pellucida. (**f**) The percentage of annexin V-positive control and NaF-treated oocytes; scale bar = 20 μm. Data are presented as mean ± SD of three independent experiments. Statistically significant differences are indicated by asterisks (*p < 0.05).

Because NaF induces DNA damage, we analyzed DNA lesions in NaF-treated oocytes. We found that phosphorylated H2AX (γH2A.X) was abundant in the nuclei of NaF-treated oocytes but was absent in the nuclei of control oocytes (Fig. 8a). Moreover, γH2A.X level markedly increased in NaF-treated oocytes compared with that in control oocytes (p < 0.05; Fig. 8b). These data suggest that NaF exposure induces DNA damage response in oocytes. Results of DNA comet assay further confirmed the occurrence of DNA damage in NaF-treated oocytes (p < 0.05; Fig. 8c,d). We next explored whether NaF exposure induced early apoptosis in oocytes by performing annexin V–FITC staining (Fig. 8e). Results of annexin V–FITC staining showed that the percentage of oocytes emitting green fluorescence was significantly higher among NaF-treated oocytes than among control oocytes (p < 0.05; Fig. 8f). These data suggest that NaF treatment triggers DNA damage response and induces autophagy and early apoptosis in porcine oocytes.

### NaF exposure affects the further development capacity of porcine oocytes

**Figure 9:**
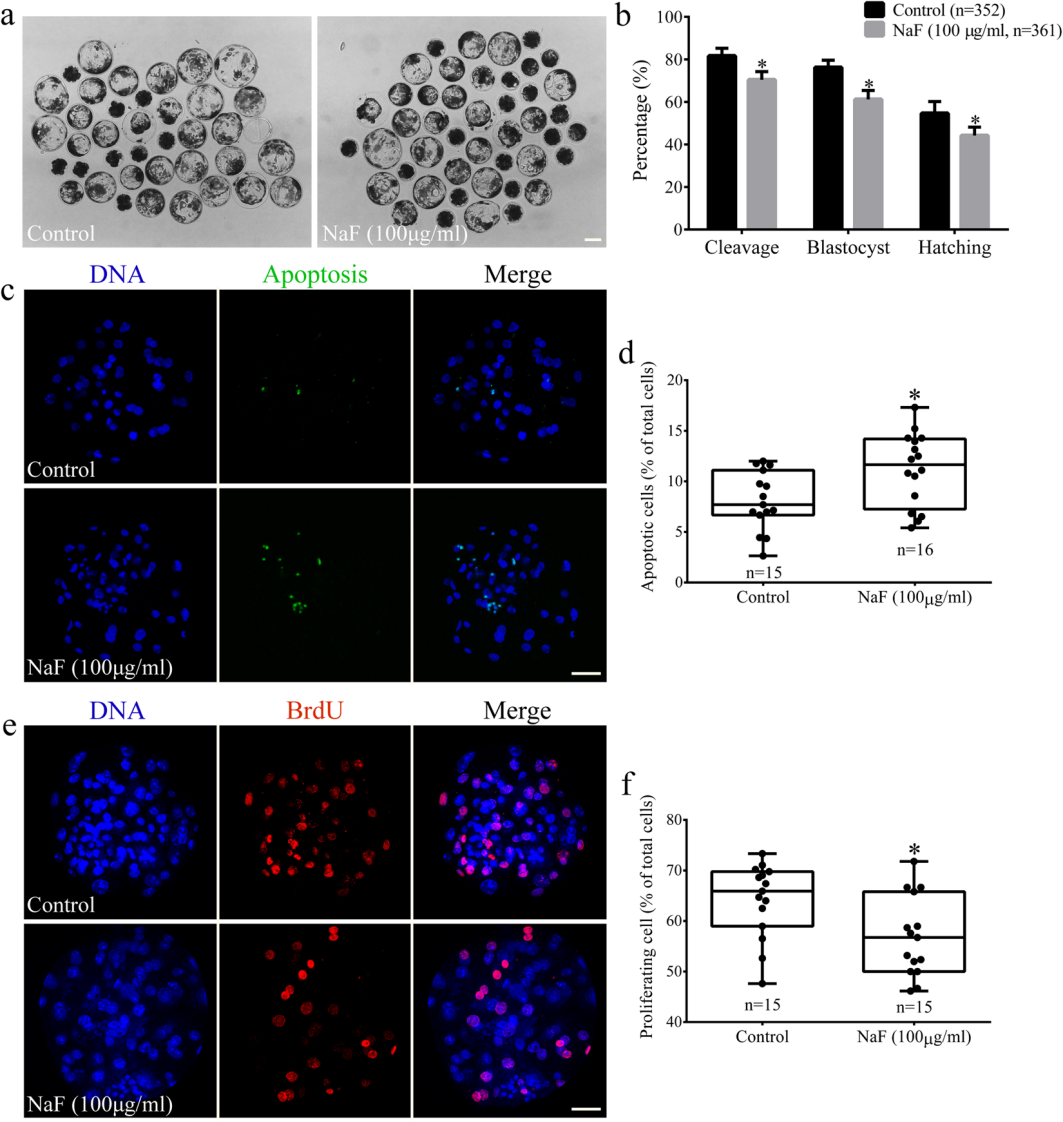
NaF exposure impairs the further developmental potential of porcine oocytes. (**a**) NaF exposure decreased the developmental potency of oocytes after parthenogenetic activation; scale bar = 100 μm. (**b**) Embryonic development rates of control and NaF-treated oocytes. (**c**) Representative images of embryos in the blastocyst stage after performing the TUNEL assay; scale bar = 50 μm. (**d**) Percentages of apoptotic cells in blastocysts developed *in vitro*. (**e**) Immunofluorescent staining of BrdU in blastocysts; scale bar = 50 μm. (**f**) Percentages of BrdU-positive cells in the blastocysts. Data are presented as mean ± SD of three independent experiments. Statistically significant differences are indicated by asterisks (*p < 0.05).

Effect of NaF exposure on the further development capacity of porcine oocytes was determined using zygotes produced by the parthenogenetic activation of MII oocytes and by monitoring their developmental potential (Fig. 9a). We found that NaF exposure disrupted early embryo development from porcine oocytes in the IVM medium (Fig. 9b). In all, 81.60% ± 3.65% control oocytes in the MII stage progressed to cleavage stage, whereas only 70.40% ± 3.85% NaF-treated oocytes in the MII stage progressed to the cleavage stage (p < 0.05). In addition, most NaF-treated oocytes in the MII stage did not reach the blastocyst stage compared with control oocytes in the MII stage (61.20% ± 4.21% vs. 76.20% ± 3.42%, p < 0.05). Moreover, NaF treatment affected subsequent blastocyst hatching rates of oocytes in the IVM medium (44.20% ± 3.96% vs. 54.60% ± 5.60%, p < 0.05). To determine the possible reason for these defects in embryo development, we assessed the apoptosis and proliferation of NaF-treated oocytes in the blastocyst stage by performing immunofluorescence staining. The percentage of apoptotic cells dramatically increased (11.48% ± 4.31% vs. 8.08% ± 2.90%, p < 0.05; Fig. 9c,d) and that of proliferating cells dramatically decreased (56.89% ± 7.90% vs. 64.15% ± 7.33%, p < 0.05; Fig. 9e,f) in blastocysts derived from NaF-treated oocytes in the IVM medium compared with that in blastocysts derived from control oocytes. Collectively, these results indicate that NaF exposure during oocyte maturation impairs the further development potential of porcine oocytes.

## Discussion

Fluorine is the most electronegative and reactive element in the periodic table. It is widely used in toothpaste and is abundantly present in natural water bodies. Oocyte maturation is a complex process, and errors during oocyte maturation prevent fertilization and block embryo development. In the present study, we investigated the effect of NaF exposure on porcine oocyte maturation. Our results showed that excessive NaF exposure during porcine oocyte maturation exerted detrimental effects on cumulus cell expansion and PBE. Excessive NaF exposure not only induced DNA damage and apoptosis in cumulus cells but also disrupted oocyte meiotic maturation by affecting cytoskeletal dynamics, oxidative stress, and apoptosis, thus affecting the embryonic development potential of porcine oocytes.

During oocyte maturation, communication between oocytes and surrounding cumulus cells and cumulus expansion play an important role. Optimum cumulus cell expansion is necessary for oocyte maturation and fertilization because it transduces signals to oocytes^30,31^. The degree of cumulus cell expansion is routinely used during IVM as a gross indicator of oocyte maturation and *in vitro* fertilization^32^. Cumulus cell expansion involves the synthesis of hyaluronic acid-rich glycosaminoglycan, which functions as a structural component of expanded cumuli and regulates oocyte maturation, in the extracellular space^33^. Our results showed that excessive NaF exposure inhibited cumulus cell expansion in a dose-dependent manner. A previous study showed that NaF exposure induced ovarian dysfunction by inducing cell apoptosis through the disruption of extracellular regulated protein kinase and c-Jun NH2 kinase signaling pathways^34^. To explore mechanisms underlying the inhibitory effect of NaF on porcine cumulus cell expansion, we determined DNA damage and apoptosis in porcine oocytes derived from COCs. We found that excessive NaF exposure increased DNA damage and apoptosis in porcine oocytes. A previous study showed that excessive long-term NaF intake induced DNA damage and apoptosis through caspase-mediated pathways in the rat kidneys^35^. Lee *et al*.^36^ investigated the relationship between cumulus cell apoptosis and oocyte quality and found that cumulus cell apoptosis was a predictor of oocyte quality and embryo outcome in oocytes cultured *in vitro*. Thus, NaF exposure may decrease PBE rate of oocytes by inducing DNA damage and apoptosis in cumulus cells. In addition, we found that excessive NaF exposure decreased GVBD rate in oocytes cultured in IVM for 24 and 30 h, suggesting that NaF altered meiotic resumption in porcine oocytes. Collectively, these data indicate that NaF acts on cumulus cells to affect porcine oocyte nuclear maturation. However, the detailed underlying molecular mechanism remains to be elucidated.

Mammalian oocytes undergo asymmetric division during meiotic maturation, and errors in meiotic maturation induce cell cycle arrest^37^. Abnormalities occurring during oocyte maturation decrease the developmental potential of oocytes, thus impairing future embryonic development^38^. Our previous study showed that excessive NaF exposure through diet decreased the quality and developmental potential of ovulated mouse oocytes *in vivo* because of meiotic disruption^19^. In mammalian oocytes, PBE is associated with normal spindle dynamics^39,40^. Results of the present study showed that excessive NaF exposure induced abnormal spindle formation in porcine oocytes, which was confirmed based on an abnormal karyotype after maturation. To divide asymmetrically, oocytes form a meiotic spindle near the cell center that subsequently moves to the cell cortex. The position of the spindle dictates the location of the contractile ring, which is responsible for dividing the cytoplasm; moreover, appropriate orientation of the spindle relative to the cell is essential for asymmetric division^41^. Movement of the spindle is driven by ATP. Studies have shown that mitochondria aggregate around the spindle during meiosis I through a dynein-mediated mechanism and migrate along with the spindle toward the cell cortex^42^. Mitochondria play a pivotal role during oocyte maturation, and mitochondrial dysfunction is suggested to induce developmental retardation and arrest of embryo^43,44^. ΔΨm is commonly used as an indicator of mitochondrial function in and viability of oocytes^45^. ΔΨm reflects the activity of hydrogen ion pump within the membrane-bound electron transport chain, which is the driving force for ATP production. In the present study, excessive NaF exposure decreased ΔΨm and ATP production in porcine oocytes. These results suggest that NaF affects the meiotic maturation of porcine oocytes by affecting mitochondrial function.

ROS disrupt mitochondrial function and play a significant role in oocyte maturation^46^. Moreover, ROS disrupt the mitochondrial membrane potential and reduce ATP production in porcine embryos^47^. Chemical exposure induces oxidative stress, which is characterized by the overproduction of free radicals, that disrupts the balance between ROS and antioxidants^48^. Porcine oocytes have higher lipid content than oocytes of other species and are highly sensitive to ROS-induced damage^49^. Our results showed that excessive NaF exposure induced excessive ROS generation in porcine oocytes. NaF exposure-induced increase in ROS level in porcine oocytes may be associated with a decrease antioxidant defense^50^. A previous study showed that sustained exposure to ROS induces mitochondrial damage^51^ and prevents the development of embryos cultured *in vitro* ^52,53^. Fluoride exposure also significantly reduces GSH activity in different cells^54–56^. Consistently, our results showed that excessive NaF exposure reduced GSH level in porcine oocytes. Cathepsin B is a prominent lysosomal cysteine protease that induces apoptosis^57^. A previous study suggested that cathepsin B promotes the translocation of apoptosis-inducing components from the mitochondria to the cytosol, thus stimulating mitochondrial membrane degradation^58^. Recent studies have suggested that cathepsin B activity is a marker of poor quality of oocytes and embryos and is associated with low developmental competence^59,60^. In the present study, increased cathepsin B activity in NaF-treated porcine oocytes further highlights the negative effect of fluoride on porcine oocyte quality.

Fluoride-induced cytotoxicity is associated with apoptosis induction^61^ and involves DNA damage^35^. Recent studies indicate that fluoride-induced apoptosis is regulated by an ROS-associated mitochondrial pathway. These findings prompted us to investigate the effects of NaF on DNA damage and apoptosis in porcine oocytes. Our results showed that excessive NaF exposure induced the accumulation of DNA damage in porcine oocytes, as determined by analyzing the level of γH2A.X, a common biomarker of cellular response for monitoring DNA damage and repair^62^. Thus, excessive NaF exposure may arrest porcine oocyte maturation by increasing the accumulation of DNA damage. A previous study showed that NaF induced the apoptosis of splenic lymphocytes^63^. Apoptosis is a complex process that may be induced by DNA damage in oocytes^64^. Our results suggest that excessive NaF exposure increases the incidence of early apoptosis. Because both DNA damage and early apoptosis affect oocyte meiotic maturation^65,66^, these might be the mechanisms through which excessive NaF exposure affects porcine oocyte meiotic maturation and blocks further development.

In conclusion, the findings of the present study indicate that exposure to NaF during IVM exerts negative effects on the quality and developmental potential of porcine oocytes by inhibiting cumulus cell expansion, disturbing cytoskeletal dynamics, and impairing nuclear and cytoplasmic maturation, thus further highlighting the deleterious effects of fluoride on reproductive systems.

## Materials and Methods

All experimental procedures were approved by the Ethics Committee of Chungbuk National University, Korea, and were conducted in accordance with institutional guidelines. All chemicals used in this study were purchased from Sigma Chemical Co. (Sigma, St. Louis, MO, USA), unless stated otherwise.

### Oocyte collection and ***in vitro*** maturation

Porcine ovaries were obtained from sows slaughtered at a local slaughterhouse. Cumulus oocyte complexes (COCs) were aspirated from the antral follicles having a diameter of 3–6 mm and were selected under a stereomicroscope. Oocytes were selected for further experiments if they had a homogeneous ooplasm and were surrounded by a minimum of three layers of cumulus cells. After washing, the COCs were transferred into an *in vitro* maturation (IVM) medium containing TCM-199 (Invitrogen, Carlsbad, CA, USA) supplemented with 10% (v/v) porcine follicular fluid, 1 μg/mL insulin, 75 μg/ml kanamycin, 0.91 mM Na pyruvate, 0.57 mM l-cysteine, 10 ng/mL epidermal growth factor, 0.5 μg/mL follicle-stimulating hormone, and 0.5 μg/mL luteinizing hormone and were cultured at 38.5 °C in an atmosphere of 5% CO_2_ and 100% humidity. Oocyte maturation was induced by culturing approximately 50 COCs in four-well dishes containing 500 μL IVM medium supplemented with various concentrations (0, 30, 60,100, and 150 μg/ml) of NaF. Before its use, NaF was dissolved in IVM medium to prepare a stock solution and was stored in the dark at −20 °C.

### Immunofluorescence staining and chromosome spreading

For immunofluorescence staining, denuded oocytes were fixed in 3.7% (w/v) paraformaldehyde for 30 min, permeabilized with 0.2% Triton X-100 in PBS containing 0.1% polyvinyl alcohol (PBS-PVA) for 1 h, and blocked with 1% bovine serum albumin (BSA) in PBS-PVA for 1 h. Next, the oocytes were incubated overnight at 4 °C with a primary antibody against γH2A.X (Cat: #2577; Cell Signaling Technology) diluted in blocking solution (dilution, 1:100). After washing extensively with PBS containing 0.1% Tween 20 and 0.01% Triton X-100 (PBS-T), the oocytes were labeled with secondary antibodies for 1–2 h at room temperature. For spindle and actin staining, the oocytes were incubated with mouse monoclonal FITC-conjugated anti-α-tubulin antibody (dilution, 1:200) and phalloidin–TRITC (dilution, 1:1000) for 1–2 h at room temperature. Finally, the oocytes were washed three times with PBS-T and were incubated with 10 μg/mL Hoechst 33342 in PBS-PVA for 10 min. After washing three times with PBS-PVA, the oocytes were mounted onto glass slides and were examined using a confocal laser scanning microscope (LSM 510 and 710 META; Zeiss, Oberkochen, Germany). The number of γH2A.X foci was evaluated using Zeiss software, as described previously^67,68^.

Chromosome spreading was performed as described previously^69^. Oocytes were treated with 1 mg/ml pronase to remove the zona pellucida and were fixed in 1% paraformaldehyde in distilled water (pH: 9.2) containing 0.15% Triton X-100 and 3 mM dithiothreitol. The samples were dried slowly at room temperature for several hours and were placed in a blocking solution for 1 h. Kinetochores were detected using anti-Bub3 antibody (anti-centromere antibody) (Cat: sc-28258; dilution, 1:50; Santa Cruz Biotechnology, USA). Chromosomes were co-stained with Hoechst 33342, and the number of chromosomes was determined using a laser scanning confocal microscope. Oocytes containing an abnormal number of chromosomes (less or more than 19) were defined as being aneuploid.

### Time-lapse microscopy for chromatin tracking

To visualize chromosomes, the oocytes were microinjected with H2B–mCherry cRNA. Images were automatically captured every 15 min for 24 h by using an inverted microscope (Lumascope 620; Etaluma Inc., Carlsbad, CA, USA) installed in an incubator with an atmosphere of 5% CO_2_ and maintained at 38.5 °C.

### Quantitative RT-PCR analysis

Total RNA was extracted from cumulus cells by using Dynabeads™ mRNA DIRECT™ Purification Kit (Cat: 61011, Invitrogen, Grand Island, NY, USA), according to the manufacturer’s instructions. Gene expression was quantified using 2^−△△Ct^ method and was normalized using the mRNA expression of *GAPDH*. PCR primers used to amplify each gene are listed in Table S1.

### Determination of mitochondrial membrane potential and ATP content

Changes in mitochondrial membrane potential (ΔΨm) were determined using MitoProbe™ JC-1 Assay Kit (Cat: M34152, Life Technologies, Carlsbad, CA). The oocytes were incubated in IVM medium containing 2 μM JC-1 for 30 min. ΔΨm was calculated as a ratio of red florescence (corresponding to activated mitochondria; J-aggregates) to green fluorescence (corresponding to less active mitochondria; J-monomers). Fluorescent signals were captured using a fluorescent microscope, and obtained images were processed using ImageJ software.

ATP content in oocytes belonging to each experimental group was measured using a commercial ATP determination kit (Cat: A22066; Life Technologies, Carlsbad, CA) based on luciferin–luciferase reaction, as described in our previous study^45^. ATP content was measured using a luminometer (Berthold, Wildbad, Germany), with a sensitivity of 0.01 pmol.

### Determination of intracellular ROS and GSH levels and cathepsin B activity

Intracellular ROS levels were determined by incubating the oocytes in the PBS-PVA medium containing 10 μM 2′,7′-dichlorodihydrofluorescein diacetate (H_2_DCFDA, Invitrogen) for 15 min. Intracellular GSH levels were determined by incubating the oocytes in the PBS-PVA medium containing 10 μM 4-chloromethyl-6.8- difluoro-7-hydroxycoumarin (CMF_2_HC, Invitrogen) for 30 min. Cathepsin B activity in the oocytes was measured using a commercial Magic Red cathepsin B assay kit (Cat: #937, Immunochemistry Technologies LLC, Bloomington, MN, USA), according to the manufacturer’s protocols. Fluorescent signals were captured using a fluorescent microscope. The same procedures, including incubation, rinsing, mounting, and imaging, were used for oocytes in all the experimental groups. Image J software was used to analyze fluorescence intensities of the oocytes.

### Comet assay and annexin V staining

Comet assays were performed using OxiSelect Comet Assay Kit (Cat: STA-350; Cell Biolabs, San Diego, CA), according to the manufacturer’s instructions. Comet tail lengths were examined in individual cumulus cell or oocyte by using a fluorescent microscope with an FITC filter (IX70; Olympus, Tokyo, Japan) and were measured using ImageJ software. Annexin V stained oocytes were detected using a commercial annexin V–FITC apoptosis detection kit (APOAF), according to the manufacturer’s protocols. Annexin V fluorescent signals were determined using a laser scanning confocal microscope.

### Parthenogenetic activation

Denuded oocytes were parthenogenetically activated using two direct-current pulses of 120 V for 60 µs in 297 mM mannitol containing 0.1 mM CaCl2, 0.05 mM MgSO4, 0.01% PVA (w/v), and 0.5 mM HEPES. Next, the oocytes were cultured in bicarbonate-buffered porcine zygote medium 5 (PZM-5) containing 4 mg/mL BSA and 7.5 µg/mL cytochalasin B for 3 h to suppress the extrusion of the pseudo-second polar body. Next, the oocytes were thoroughly washed and cultured in four-well plates containing bicarbonate-buffered PZM-5 supplemented with 4 mg/mL BSA at 38.5 °C and in 5% CO_2_ for 7 days without changing the medium.

### Cell proliferation and apoptosis assays

Proliferation and apoptosis of cells in blastocysts were assessed by performing BrdU^70^ and TUNEL assays, respectively, as described previously, by using the following chemicals, antibodies, and kits: BrdU (100 µM), anti-BrdU monoclonal antibody (B2531; dilution, 1:10), rabbit anti-mouse IgG Alexa Fluor 568-conjugated polyclonal antibody (Cat: A11061; dilution, 1:100; Invitrogen), fluorescein-conjugated dUTP, and terminal deoxynucleotidyl transferase enzyme (*In Situ* Cell Death Detection Kit; Roche; Mannheim, Germany).

### Statistical analysis

Data obtained in percentages were transformed using arcsine transformation before performing statistical analysis and are presented as mean ± SD. Data obtained from the experimental groups were compared using Student’s *t*-test. All statistical analyses were performed using GraphPad Prism 6.01 (GraphPad Software, San Diego, CA). p < 0.05 was considered statistically significant.

## Electronic supplementary material

Supplementary Material

Supplementary Video s1

Supplementary Video s2
