# Supplementary material for: Sodium fluoride exposure exerts toxic effects on porcine oocyte maturation
Source: Sci Rep. 2017 Dec 6;7:17082. doi: 10.1038/s41598-017-17357-3 (PMC5719058; doi:10.1038/s41598-017-17357-3)
Supplement: Supplementary file 1 — Supplementary Material [file 41598_2017_17357_MOESM1_ESM.pdf]

Supplementary Material

Sodium fluoride exposure exerts toxic effects on porcine oocyte maturation

Shuang Liang [1, 2, #], Zheng-Wen Nie [1, #], Minghui Zhao [1, #], Ying-Jie Niu [1], Kyung-Tae Shin [1], and Xiang-Shun Cui [1, *]

[1] Department of Animal Science, Chungbuk National University, Cheongju, Chungbuk, 361-763, Republic of Korea.

[2] Department of Laboratory Animal Center, College of Animal Sciences, Jilin University, Changchun, 130062, China.

[#] Shuang Liang, Zheng-Wen Nie and Minghui Zhao contributed equally to this work.

[*] Corresponding Author. Xiang-Shun Cui (e-mail: xscui@cbnu.ac.kr.)

Figure s1. Cumulus cell expansion grade of porcine oocytes was evaluated at 44 h. Representative images of porcine COCs showing four grades of cumulus cell expansion: (a) Grade 1, no expansion; (b) Grade 2, expansion only in the outer cumulus cell layers; (c) Grade 3, expansion of all cumulus cell layers, except the corona radiata; (d) Grade 4, expansion of all cumulus cell layers, including the corona radiata. Scale bar = 50 µm.

Supplementary video s1 and s2. Time-lapse video of control (video s1) and NaF-treated oocytes (video s2) microinjected with H2B–mCherry cRNA. The control oocyte microinjected with H2B–mCherry cRNA reached the MII stage at the end of culture period (40 h), whereas the NaF-treated oocyte microinjected with H2B–mCherry cRNA did not reach the MII stage at the end of the culture period. Red, H2B–mCherry.

Fig. s1

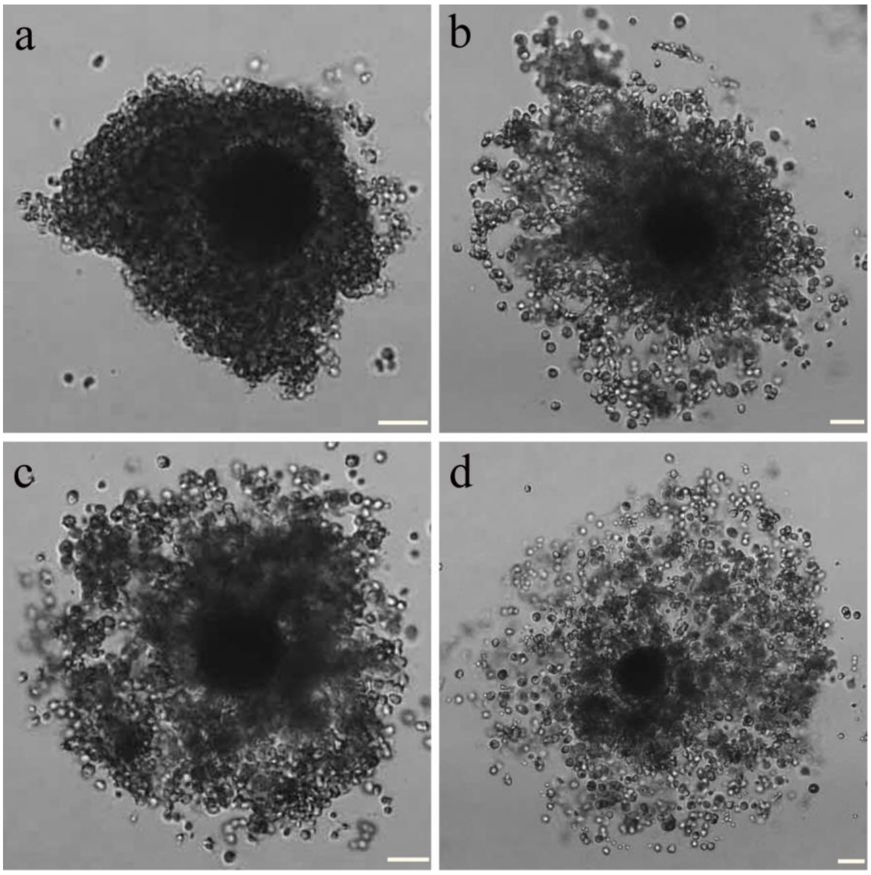

Table s1 Primer sequences used in real-time RT-PCR

Gene	Primer sequence (5’-3’)	Product size (bp)	Genbank number
BCL2	F:GGCTGCGGGAACATAATAGA<br>R:GCAGCTCTGGGTCAAACTTC	*193*	NM_214285
BAX	F:AGGGCGATGAACCACAGT<br>R:GCTCCAGCGAACGACAAT	*111*	XM_005664710.2
CASP3	F:GGTACCACTATGGGCTCCTCAC<br>R:TCGGCCCTTACCGCTTCT	*110*	NM_214131.1
GAPDH	F: TTCCACGGCACAGTCAAG<br>R: ATACTCAGCACCAGCATCG	*117*	NM_001206359

F, forward; R, reverse.
